# CALL FOR FOUNDING MEMBERS OF THE EUROPEAN ORGANIZATION FOR UNIVERSITY PHYSICAL AND REHABILITATION MEDICINE (UNIPRM)

**DOI:** 10.2340/jrm.v57.44520

**Published:** 2025-09-04

**Authors:** Stefano NEGRINI, Xavier De BOISSEZON, Gerold STUCKI, Jari AROKOSKI, Katja Groleger SRŠEN, Sofie RUMMENS, Henk STAM, Alessandro PICELLI, Francesca GIMIGLIANO

**Affiliations:** 1 Department of Biomedical, Surgical, and Dental Sciences, University of Milan “La Statale”, Milan; 2 IRCCS Galeazzi S. Ambrogio Hospital, Milan, Italy; 3 Department of Physical Medicine and Rehabilitation, University Hospital of Toulouse, Toulouse; 4 ToNIC, NeuroImaging Center, University of Toulouse, Inserm, UPS, Toulouse, France; 5 Faculty of Health Sciences and Medicine, University of Lucerne, Lucerne; 6 Center for Rehabilitation in Global Health Systems, WHO Collaborating Center, University of Lucerne, Lucerne; 7 Swiss Paraplegic Research, Nottwil, Switzerland; 8 Department of Internal Medicine and Rehabilitation Medicine, Division of Rehabilitation Helsinki University Central Hospital and University of Helsinki, Helsinki, Finland; 9 Department for Rehabilitation, University Rehabilitation Institute of Republic Slovenia – Soča; 10 University Ljubljana, Medical Faculty, Ljubljana, Slovenia; 11 Department of Development and Regeneration, KU Leuven - University of Leuven, Leuven, Belgium; 12 Department of Rehabilitation Medicine Erasmus MC, University Medical Center, Rotterdam, The Netherlands; 13 Department of Neurosciences, Biomedicine and Movement Sciences, University of Verona, Verona; 14 Department of Mental and Physical Health and Preventive Medicine, University of Campania “Luigi Vanvitelli”, Naples, Italy

**Keywords:** academic medical centres, clinical competence, curriculum, faculty, medical, health workforce, interdisciplinary communication, leadership, teaching

## Abstract

Rehabilitation is increasingly recognized as a key component of health systems worldwide. To meet the growing demand for rehabilitation services, it is essential to strengthen academic capacity in Physical and Rehabilitation Medicine within universities. Academic structures are critical for training future physicians and professionals, and for advancing research and innovation in rehabilitation. In response to this need, a new European organization has been established: the European Organization of University Physical and Rehabilitation Medicine. This organization aims to unite and support university-based educators and researchers in the field, fostering excellence in teaching, research, clinical care, and academic leadership. The initiative has been developed through collaboration with major European and international rehabilitation bodies and is grounded in the values of representativity, inclusiveness, and collaboration. The organization invites current and future university professors in Physical and Rehabilitation Medicine to join as founding members and contribute to shaping its mission, structure, and strategic direction. By building a strong academic network across Europe, this initiative seeks to reinforce the foundations of rehabilitation in health systems and promote the development of a skilled and innovative workforce. This commentary outlines the rationale, development, and goals of the new organization and calls for broad participation from the academic community.

## LAY ABSTRACT

Rehabilitation helps people regain and maintain their health and independence after illness, injury, or disability. To meet the growing need for rehabilitation, we must ensure that future doctors and specialists are well trained. This requires strong teaching and research programmes in universities. A new European organization called the European Organization of University Physical and Rehabilitation Medicine has been created to support this goal. It brings together university teachers and researchers from across Europe who are dedicated to improving rehabilitation education, research, and care. The organization also supports young professionals who want to become university professors in this field. By working together, members aim to build a stronger academic foundation for rehabilitation, which is essential for better health systems and better care for people in need.

The World Health Assembly resolution on “Strengthening Rehabilitation in Health Systems” [1], together with the WHO’s Rehabilitation 2030 Initiative [2], emphasizes the urgent need to build rehabilitation workforce capacity [3]. Achieving this goal requires robust academic structures within universities. In response, leading Physical and Rehabilitation Medicine (PRM) organizations – including the International Society of Physical and Rehabilitation Medicine (ISPRM), the European Academy of Rehabilitation Medicine (EARM), and the Association of Academic Physiatrists (AAP) – have jointly called for efforts to strengthen academic capacity in PRM [4]. This is essential to reinforce the foundations of the specialty, attract a stronger workforce, and ensure future physicians, PRM specialists, and rehabilitation professionals are equipped with appropriate knowledge and skills.

To advance this effort in Europe and building on the arguments presented in the paper “Why we need a representative organization of academic PRM in Europe, and why we need it now” [4], its authors have initiated the creation of the European Organization of University Physical and Rehabilitation Medicine (UniPRM).

Following an initial meeting held on 7 January 2025, in Milan, the foundational steps included the establishment of a Founding Council (comprising 1 invited university professor per European country) on 10 and 12 February 2025 (see Table SI for current membership), and a Founding Executive Board on April 2, 2025 (see Table SII for membership). To date, 37 countries have expressed interest in joining the organization through their designated Council members. The Founding Executive Board and Council now extend an open invitation to all current and prospective PRM professors across Europe to join UniPRM as Founding Members. By doing so, members will actively contribute to shaping the organization from its inception. This includes defining its vision, mission, and strategic plan for 2030, as well as its governance and financing models. Founding Members will also help establish the working structure across the 4 pillars of academic PRM: education, research, clinical care, and academic leadership and administration.

## WHAT IS UNIPRM?

UniPRM is a representative, inclusive, and collaborative organization dedicated to strengthening academic capacity in PRM across European universities. Academic capacity is essential to ensure that future physicians, PRM specialists, and rehabilitation professionals are well trained to meet the growing rehabilitation needs within health systems. UniPRM serves as the academic voice of PRM in European universities, providing a platform to connect and empower academic PRM communities to meet this challenge together. Its mission is to unite and advance academic PRM by fostering excellence in education, research, clinical care, and academic leadership.

UniPRM is grounded in 3 core values:

*Representativity*: Every existing and future national organization of PRM professors is represented on the UniPRM Council, ensuring broad geographic and institutional inclusion across Europe.

*Inclusiveness*: All current and prospective professors teaching PRM in European universities are welcome to join UniPRM through an open application process, fostering individual engagement, diversity, and academic collaboration.

*Collaboration*: UniPRM actively seeks partnerships with other relevant organizations in Europe and internationally. It aims to work closely with the AAP, the EARM, the European Union of Medical Specialists (UEMS) PRM Section and Board, the European Society of Physical and Rehabilitation Medicine (ESPRM), and the ISPRM.

## OUR NAME AND IDENTITY

The name UniPRM stands for University Physical and Rehabilitation Medicine, emphasizing the organization’s foundation in university-based academic PRM. The name was carefully developed over a 3-month consultation process involving the Founding Executive Board and their academic networks, and the presidents and executive committees of the European Alliance of PRM, including EARM, ESPRM, and the UEMS PRM Section and Board.

The final choice reflects 3 key elements:

- The academic discipline: the term University PRM highlights the organization’s focus on academic PRM as anchored in universities.
- The geographical scope: European defines the regional focus of the initiative.
- The organizational form: The term “Organization” was selected as the most appropriate to represent UniPRM’s goals because:
  - it encompasses the development of academic PRM across its 4 pillars: education, research, clinical care, and academic leadership;
  - it provides a flexible and future-oriented structure, while remaining firmly rooted in its academic mission;
  - it is distinct and not currently used by any other European or international PRM body.

## WHO CAN JOIN UNIPRM?

UniPRM welcomes a broad spectrum of academic professionals (both fully and partially employed by universities) who are committed to advancing PRM. Membership is open to individuals across different stages of their academic careers, including:

- *Full Professors of PRM (grade A)*
  Individuals holding the highest academic rank in their country, typically affiliated with a medical faculty or medical school.
- *Associate Professors of PRM (Grade B)*
  Academics at the second-highest rank, also affiliated with medical faculties or medical schools.
- *Prospective Professors of PRM (Grade C)*
  PRM trainees and physicians pursuing an academic career with the goal of becoming associate or full professors. This category is particularly important, as one of UniPRM’s core missions is to strengthen the academic PRM workforce by supporting the next generation of university-based PRM educators and researchers.
- *University-affiliated Academics in PRM*
  PRM physicians engaged in teaching and research within universities, including those working outside traditional medical faculties.

## BECOME A FOUNDING MEMBER: HOW TO JOIN

This is a unique opportunity to help shape the foundation of UniPRM as a Founding Member. Founding Members will play a key role in defining the organization’s strategic direction, structure, and activities from the outset.

If you are interested in joining UniPRM as a Founding Member, please send an email to universityPRM@gmail.com, copying in the Founding Council member of your Country (see Table SII), and the chair of your national organization of university PRM professors, if such an organization exists.

In your message, please include the following information:

- Name
- Country
- University
- Academic role, as defined above in the membership categories (e.g., Full Professor, Associate Professor, Prospective Professor, University-affiliated academic).

For more information or guidance, you are encouraged to contact the Founding Council member representing your Country.

## JOIN THE FIRST SCIENTIFIC SYMPOSIUM OF UNIPRM

We are pleased to invite all founding Members to the first scientific symposium of UniPRM, which will take place on Friday, 14 November 2025, at the University Campus of the University of Lucerne in Nottwil, Switzerland.

This event marks a key milestone in the development of UniPRM and offers a unique opportunity to engage with colleagues from across Europe who are committed to advancing academic PRM.

### How to register

To register, please send an email to gerold.stucki@paraplegie.ch, with cc to universityPRM@gmail.com.

In your message, kindly include the following information:

- Name
- Country
- University
- Academic role (see membership categories)
- Attendance mode: on-site in Nottwil (accommodation is available at Hotel Sempachersee and in the broader Lucerne region) or via live stream

## JOIN US IN SHAPING THE FUTURE OF ACADEMIC PRM

We warmly invite all current and future PRM professors in Europe to join UniPRM as Founding Members and become part of a growing community dedicated to advancing academic PRM. This is a unique opportunity to contribute to the strategic direction, structure, and activities of UniPRM from the very beginning.

Our shared vision is to establish PRM as a fully recognized academic and clinical discipline, a cornerstone of modern medicine. PRM should lead innovation in human functioning, rehabilitation, and integrated care across health systems. Through strong academic structures in every European university, UniPRM aims to foster excellence in education, research, clinical care, and academic leadership. By joining UniPRM, you will help build the academic foundation that is essential to strengthening rehabilitation within health systems across Europe.

## Supplementary Material



## References

[1] World Health Organization. Resolution: Strengthening rehabilitation in health systems; 2023 [cited 2025, Apr 19]. Available from: https://apps.who.int/gb/ebwha/pdf_files/EB152/B152(10)-en.pdf

[2] Gimigliano F, Negrini S. The World Health Organization “Rehabilitation 2030: a call for action”. Eur J Phys Rehabil Med 2017; 53: 155–168. 10.23736/S1973-9087.17.04746-3 28382807

[3] Sivan M, Negrini S. An expanded workforce is needed to strengthen rehabilitation in health systems. BMJ 2024; 384: q60. 10.1136/bmj.q60 38212055

[4] Frontera WR, Stucki G, Engkasan JP, Francisco GE, Gutenbrunner C, Hasnan N, et al. Advancing academic capacity in physical and rehabilitation medicine to strengthen rehabilitation in health systems worldwide: a joint effort by the European Academy of Rehabilitation Medicine, the Association of Academic Physiatrists, and the International Society of Physical and Rehabilitation Medicine. Am J Phys Med Rehabil 2022; 101: 897–904. 10.1097/PHM.0000000000002067 35777886 PMC9377495

[5] Stucki G, Stam HJ, Gimigliano F, Negrini S. Why we need a representative organization of academic PRM in Europe, and why we need it now. J Rehabil Med 2025; 57: jrm42369. 10.2340/jrm.v57.42369 39749426 PMC11680898

