# Supplementary material for: CALL FOR FOUNDING MEMBERS OF THE EUROPEAN ORGANIZATION FOR UNIVERSITY PHYSICAL AND REHABILITATION MEDICINE (UNIPRM)
Source: J Rehabil Med. 2025 Sep 4;57:44520. doi: 10.2340/jrm.v57.44520 (PMC12421331; doi:10.2340/jrm.v57.44520)
Supplement: Supplementary file 1 [file JRM-57-44520-s1.pdf]

Supplementary material has been published as submitted. It has not been copyedited, or typeset by Journal of Rehabilitation Medicine

Table SI. UniPRM Founding Executive Board

Voting members		
Founding President	Stefano Negrini (ITA)	stefano.negrini@unimi.it
Founding President-elect	Xavier de Boissezon (FRA)	deboissezon.xavier@chu-toulouse.fr
Founding Chair of the Council	Francesca Gimigliano (ITA)	francesca.gimigliano@unicampania.it
Founding Full Professors (grade A) representative	Jari Arokoski (FIN)	jari.arokoski@hus.fi
Founding Associate Professors (grade B) representative	Katja Groleger Sršen (SVN)	katja.groleger@mf.uni-lj.si
Founding Prospective Professors (grade C) representative	Sofie Rummens (BEL)	sofie.rummens@uzleuven.be
**Nonvoting members**		
Founding advisor (promoter)	Gerold Stucki (CHE)	gerold.stucki@paraplegie.ch
Founding advisor (promoter)	Henk Stam (NLD)	hendrikjanstam@gmail.com
Secretary	Alessandro Picelli (ITA)	alessandro.picelli@univr.it
Treasurer	François Constant Boyer (FRA)	fboyer@chu-reims.fr

Table SII. UniPRM Founding Council Board

COUNTRY	REPRESENTATIVE	ADDRESS
Andorra	Merce Avellanet	merceavellanet@gmail.com
Austria	Richard Crevenna	richard.crevenna@meduniwien.ac.at
Federation of Bosnia and Herzegovina	Mirsad Muftic	mhs@bih.net.ba
Bulgaria	Elena Ilieva	elena_md@yahoo.com
Czech Republic	Alena Kobesova	alena.kobesova@lfmotol.cuni.cz
Croatia	Tonko Vlak	tonkovlak@gmail.com
Cyprus	Nicolas Christodoulou	nicolas.christodoulou.cyprus@gmail.com
Estonia	Aet Lukmann	Aet.Lukmann@kliinikum.ee
Finland	Jaro Karppinen	jaro.karppinen@oulu.fi
France	François Rannou	francois.rannou@aphp.fr
Germany	Christoph Gutenbrunner	christoph.gutenbrunner@gmail.com
Greece	Markos Sgantzos	sgantzos@med.uth.gr
Hungary	Gabor Fazekas	fazekas123@t-online.hu
Ireland	Raymond Carson	Raymond.Carson@NRH.IE
Italy	Pietro Fiore	pietro.fiore@unifg.it
Kosovo	Ardiana Murtezani	ardiana.murtezani@uni-pr.edu
Latvia	Anita Vetra	anita.vetra@yahoo.com
Lithuania	Alvydas Juocevicious	alvydas.juoce@gmail.com
Malta	Andrei Agius Anastasi	aagi0017@um.edu.mt
Moldova	Oleg Pascal	oleg.pascal@usmf.md

Montenegro	Vesna Bokan Mirković	vesnabokanmir@gmail.com
Netherlands	Alexander Geurts	Sander.Geurts@radboudumc.nl
North Macedonia	Koevska Valentina	valeskoevska@yahoo.com
Norway	Cecilie Roe	eller@medisin.uio.no
Poland	Elżbieta Miller	elzbieta.dorota.miller@umed.lodz.pl
Portugal	Joao Pinheiro	reabmedica@hotmail.com
Republic of Sprska	Tatjana Nozica Radulović	tatjana.nozica@med.unibl.org
Romania	Mihai Berteanu	mberteanu@gmail.com
Serbia	Milica Lazovic	lazovicmilica15@gmail.com
Slovakia	Karol Hornacek	hornacek59@gmail.com
Slovenia	Helena Burger	helena.burger@ir-rs.si
Spain	Soraya Hijazi Vega	soraya.hijazi.vega@gmail.com
Sweden	Katharina Stibrant-Sunnerhagen	ks.sunnerhagen@neuro.gu.se
Switzerland	Anke Scheel	anke.scheel-sailer@paraplegie.ch
Turkey	Ayse A. Küçükdeveci	ayse.kucukdeveci@gmail.com
United Kingdom	Manoj Sivan	M.Sivan@leeds.ac.uk
Ukraine	Oleksandr Vladimirov	avladimirov05@gmail.com
